# Practice of radiation therapy for anal cancer in Austria—a survey on behalf of the Austrian radiation oncology society gastrointestinal tumor group (ÖGRO-GIT)

**DOI:** 10.1007/s00066-021-01842-w

**Published:** 2021-09-30

**Authors:** S. Gerum, W. Iglseder, R. Schmid, K. Peterka, T. H. Knocke-Abulesz, P. Harl, S. Schwaiger, I. Reiter, J. Salinger, C. Venhoda, G. Kurzweil, M. Poetscher, R. Jaeger, B. Celedin, P. Clemens, F. Roeder

**Affiliations:** 1 Universitätsklinik für Radiotherapie und Radio-Onkologie, Landeskrankenhaus Salzburg, Uniklinikum der Paracelsus Medizinischen Universität, Müllner Hauptstraße 48, 5020 Salzburg, Austria; 2 Universitätsklinik für Radioonkologie, Medizinische Universität Wien, Universitätsklinikum AKH Wien, Comprehensive Cancer Center Vienna, Währinger Gürtel 18-20, 1090 Wien, Austria; 3 Institut für Radioonkologie, Kaiser-Franz-Josef-Spital/SMZ Süd-Klinik Favoriten, Kundratstraße 3, 1100 Wien, Austria; 4 Sonderabteilung Strahlentherapie, Wiener Gesundheitsverbund Klinik Hietzing, Wolkersbergenstraße 1, 1130 Wien, Austria; 5 Institut für Radioonkologie, SMZ – Ost Donauspital der Stadt Wien, Langobardenstraße 122, 1220 Wien, Austria; 6 Institut für Radioonkologie, Klinik Ottakring, Wilhelminenspital der Stadt Wien, Montleartstraße 37, 1160 Wien, Austria; 7 Institut für Radioonkologie und Strahlentherapie, Landesklinikum Wiener Neustadt, Corvinusring 3–5, 2700 Wiener Neustadt, Germany; 8 Klinische Abteilung für Strahlentherapie – Radioonkologie, Universitätsklinikum Krems, Karl Landsteiner Privatuniversität für Gesundheitswissenschaften, Mitterweg 10, 3500 Krems an der Donau, Austria; 9 Klinik für Radioonkologie, Klinikum der Barmherzigen Schwestern, Ordensklinikum Linz, Seilerstätte 4, 4010 Linz, Austria; 10 Klinik für Radioonkologie/Strahlentherapie, Salzkammergutklinikum Vöcklabruck, Dr.-Wilhelm-Boch-Straße 1, 4840 Vöcklabruck, Austria; 11 Universitätsklinik für Strahlentherapie – Radioonkologie, Comprehensive Cancer Center Graz, Medizinische Universität Graz, Auenbruggerplatz 32, 8036 Graz, Austria; 12 Universitätsklinik für Strahlentherapie – Radioonkologie, Medizinische Universität Innsbruck, Anichstraße 35, 6020 Innsbruck, Austria; 13 Institut für Strahlentherapie/Radioonkologie, Klinikum Klagenfurt am Wörthersee, Feschnigstraße 11, 9020 Klagenfurt, Austria; 14 Institut für Radioonkologie und Strahlentherapie, Landeskrankenhaus Feldkirch, Carinagasse 47, 6807 Feldkirch, Austria

**Keywords:** Anal cancer, Chemoradiation, Survey, Guidelines, Nationwide

## Abstract

**Purpose:**

We conducted a patterns-of-care survey on chemoradiation for locoregionally confined anal cancer in Austria to evaluate areas of disagreement and to identify possible targets for further standardization.

**Methods:**

An anonymous questionnaire comprising 38 questions was sent to all Austrian radiation oncology departments. Results were analyzed descriptively and compared to two international guidelines.

**Results:**

The response rate was 93%. Work-up generally includes DRE, endoscopy, and cross-sectional imaging of chest/abdomen and pelvis. PET-CT is used by 38%. Screening for HIV and biopsies of suspicious lymph nodes are infrequently used. All centers perform IMRT, mainly with daily IGRT. Median doses to the primary are 54.7 Gy (T1–2) and 59.4 Gy (T3–4). Suspicious nodes receive a boost (median dose 54 Gy), while elective nodal areas are mainly treated with 45–50.4 Gy. Target delineation of elective nodal areas seems generally uniform, although disagreement exists regarding inclusion of the common iliac nodes. No agreement was found for OAR-delineation and dose constraints. Concurrent chemotherapy is mitomycin and 5‑FU/capecitabine. Supportive care beyond skin care is infrequently offered. Intensive follow-up is performed for at least 5 years. Treatment of T1N0 shows considerable disagreement.

**Conclusion:**

We found a high rate of agreement between the centers and concordance with major guidelines. PET-CT, routine HIV testing, and biopsies of suspicious LN seem underrepresented. The largest controversy regarding target volumes concerns inclusion of the common iliac nodes. Prescribed doses are generally in line with the recommendations or higher. OAR delineation, dose constraints, supportive care, and treatment of early anal cancer represent areas for further standardization.

**Supplementary Information:**

The online version of this article (10.1007/s00066-021-01842-w) contains supplementary material, which is available to authorized users.

## Background

Definitive chemoradiation (CRT) is the standard of care for curative-intent treatment of locoregionally confined anal cancer (AC). While the general indication is widely accepted, international consensus for issues like staging procedures; radiation technique, dose, and fractionation; target volume definition; supportive care; patient counseling; and treatment of early lesions is heterogeneous. Since not all pending questions will be addressed in prospective trials, evidence of lower levels must be taken into account. Surveys generally offer an easy possibility to analyze patterns of care; however, their value is often limited by low response rates. In this regard, regions with a limited number of centers treating the majority of patients may offer some advantages. In Austria, access to radiation therapy is limited to 14 institutions, which can be addressed easily via the *Östereichische Gesellschaft für Radio-Onkologie* (ÖGRO; Austrian Society of Radiation Oncology [RO]). We therefore conducted a survey regarding CRT of locoregionally confined AC to compare actual patterns of care in Austria with the recommendations of major international guidelines. The following text represents a summary of the main issues, while the full text including detailed results and discussion is available as electronic supplementary material.

## Methods

All 14 Austrian RO institutions were invited to take part in the survey on the regular treatment of squamous cell, locoregionally confined, non-metastatic AC. It included 38 questions with either a predefined choice of answers or space for written description of the center’s approach (supplementary material). Because of the known controversies in treating early-stage AC, we included a clinical example and asked for the center’s opinion regarding the optimal approach for T1N0 lesions (supplementary material), while all other questions were restricted to T2-4N0-1M0 stages. The survey was made available online to allow anonymous participation. Numerical variables were analyzed descriptively. Results were compared to the current versions of international, multinationally used multidisciplinary guidelines from the US [1] and Europe [2]. A comparison with other national guidelines (e.g., German, French, or British guidelines) was intentionally not performed.

## Results

### Response rate/general information

Response rate was 93% (13/14 centers). Most institutions (69%) operate 1–3 linear accelerators, only one center is equipped with > 6. The median number of patients treated with curative-intent (chemo)radiation per year and institution was 14 (7–35). Written standard operating procedures are available in 46%, while no center reported clinical trials currently recruiting patients.

### Work-up

Procedures routinely used for diagnostic work-up are listed in Table 1. Clinical examination is performed in all centers, although only 85% explicitly reported digital rectal examination (DRE). All perform some form of endoscopy with histological confirmation and pelvic MRI for locoregional staging. Endorectal ultrasound (39%), ultrasound of inguinal lymph nodes (LN) (23%), or biopsies of suspicious inguinal LN (15%) are infrequently used. To rule out distant metastases, all centers perform some form of chest and abdominal imaging; however, PET-CT is routinely used in only a minority (38%). Routine HIV testing is done in only two centers (15%) and does not change the general treatment principles. Routine HPV testing of the pathological specimen of the primary tumor is performed in 54%, although altering the treatment regime in only one center. Female patients generally receive a specific gynecological clinical examination in 62% and only in case of suspected vaginal involvement in a further 15%. Male patients wishing to preserve fertility or female patients with childbearing potential are routinely counselled with regard to fertility protection or cryopreservation in 77%. Multidisciplinary discussion of the case prior to treatment is routinely done in 85%.

**Table 1:** Examinations for work-up

Examination	*n*	%
Pelvic MRI	13	100
Procto-/rectoscopy	12	92
DRE	11	85
Abdominal CT	11	85
Gynecologic examination^a^	10	77
Counseling (fertility protection)	10	77
Chest CT	9	69
Colonoscopy	7	54
HPV status (biopsy)	7	54
Endorectal ultrasound	5	39
PET-CT	5	39
Inguinal ultrasound	3	23
Biopsy of suspicious inguinal nodes	2	15
HIV status	2	15
Chest X‑ray	1	8
Abdominal ultrasound	1	8
Tumor marker (SCC)	1	8

*n* number of centers, *%* percentage of centers, *MRI* magnetic resonance imaging, *DRE* digital rectal examination, *CT* computed tomography, *HPV* human papilloma virus, *PET-CT* positron-emission computed tomography, *HIV* human immunodeficiency virus, *SCC* squamous cell carcinoma antigen

^a^in female patients

### Simulation/treatment planning

Treatment planning CT is done in prone position in one institution, while the remaining centers (92%) prefer supine position. Oral or intravenous contrast agents are used in 8 and 31%, respectively. Patients are required to have a full bladder in all institutions (100%) and eight centers (62%) also advise an empty rectum. The lower edge of the primary tumor (PT) or the anal verge is specifically marked with radio-opaque material in 77%, while only 23% of the centers mark the vagina in female patients. Four institutions (31%) indicated the use of bolus material in case of a prolapsed primary.

### Target volume definition/prescription dose

In node-negative cases, all centers include the bilateral inguinal and internal iliac nodes into the CTV, most centers (85–92%) also include the external iliac, mesorectal, presacral, and obturator nodes, but only a minority (46%) include the common iliac nodes (Table 2). Indicated total doses were 30.6–54 Gy in conventional fractionation (single dose 1.65–2.0 Gy), with the majority of centers (*n* = 7) using total doses of 45–50.4 Gy for all elective nodal regions using 1.8 Gy per fraction.

**Table 2:** Elective nodal volumes and prescription doses in cN0 patients

Elective nodal region	*n*	%	Total dose (Gy)	Single dose (Gy)
Bilateral inguinal nodes	13	100	45 (30.6–50.4)	1.8 (1.65–2)
Bilateral internal iliac nodes	13	100	46 (30.6–50.4)	1.8 (1.65–2)
Bilateral external iliac nodes	11	85	47.3 (39.6–50.4)	1.8 (1.65–2)
Bilateral common iliac nodes	6	46	47.9 (39.6–50.4)	1.8 (1.65–2)
Mesorectal nodes	12	92	49.5 (30.6–54)	1.8 (1.7–2)
Presacral nodes	12	92	46.4 (30.6–50.4)	1.8 (1.7–2)
Bilateral obturator nodes	12	92	47.3 (39.6–50.4)	1.8 (1.6–2)

*cN0* clinically node-negative case, *n* number of centers who would include the region, *%* percentage of centers who would include the region, total dose: median total dose for this region (range of doses for this region), *single dose* median single dose for this region (range of single doses for this region), if a center specified a dose range, the mean of the dose range was used for calculation of the median values

In cN+ cases, all centers include the bilateral inguinal, internal iliac, external iliac, mesorectal, and obturator LN into the CTV. All but one (92%) would also include the presacral and 62% the common iliac nodes (Table 3). Indicated total doses were 30.6–60 Gy in conventional fractionation (single doses 1.65–2.0 Gy), although the median total doses were slightly higher for all elective nodal regions compared to the cN0 patients. Again, the majority of centers (*n* = 8) indicated total doses of 45–50.4 Gy for all elective nodal regions in 1.8-Gy single doses.

**Table 3:** Elective nodal volumes and prescription doses in cN+ patients

Elective nodal region	*n*	%	Total dose (Gy)	Single dose (Gy)
Bilateral inguinal nodes	13	100	50 (30.6–60)	1.8 (1.7–2)
Bilateral internal iliac nodes	13	100	50.4 (30.6–50.4)	1.8 (1.7–2)
Bilateral external iliac nodes	13	100	50.4 (30.6–50.4)	1.8 (1.65–2)
Bilateral common iliac nodes	8	62	50.2 (45–50.4)	1.8 (1.65–2)
Mesorectal nodes	12	92	50.4 (30.6–54)	1.8 (1.7–2)
Presacral nodes	13	100	50.2 (30.6–54)	1.8 (1.7–2)
Bilateral obturator nodes	13	100	50.4 (30.6–50.4)	1.8 (1.7–2)

*cN+* clinically node-positive case, *n* number of centers who would include the region, *%* percentage of centers who would include the region, *total dose* median total dose for this region (range of doses for this region), *single dose* median single dose for this region (range of single doses for this region), if a center specified a dose range, the mean of the dose range was used for calculation of the median values

Most centers (77%) increase the total dose (boost) in suspicious nodes up to a median dose of 54 Gy (range 50–60 Gy), mainly independent of LN size. All institutions used conventional or slightly accelerated fractionation for boosting the LN or LN area (single doses 1.8–2.2 Gy).

Dose escalation in the PT region is generally performed by 92% (*n* = 12), either as sequential (54%) or simultaneous integrated (15%) external beam photon boost, as electron boost (15%), or via brachytherapy (15%). For small tumors (cT1–2), only 85% generally prescribe a boost, while all prescribe a boost for larger primaries (cT3–4). Ten centers (77%) explicitly indicated increased total doses in cT3–4 compared to cT1–2 tumors. For cT1–2 primaries, a median total dose of 54.7 Gy (50.4–59.4 Gy, single doses 1.8–2.2 Gy) was prescribed, while larger tumors (cT3–4) receive a median total dose of 59.4 Gy (55–64.4 Gy, single doses 1.8–2.3 Gy).

### Treatment procedure

All centers use volumetric intensity-modulated RT. Participants were asked to provide information on generally outlined organs at risk (OAR) and whether they use specific dose constraints or just try to keep the dose reasonably low during the planning process (see Table 4). The given dose constraints varied widely even for a single organ at risk (data not shown).

**Table 4:** Outlined organs at risk (OAR) and use of specific dose constraints

	Outlined		Specific constraints	
Organ at risk	*n*	%	*n*	%
Bladder	13	100	10	77
Femoral head	10	77	8	61
Small bowel	9	69	5	38
Bowel bag	8	61	6	46
Colon	5	38	1	8
External genitalia	5	38	3	23
Cauda equina	2	15	2	15
Pelvic bone	1	8	0	0

*n* number of centers, *%* percentage of centers

Some form of image-guided RT (IGRT) is performed by all centers. Most departments indicated daily imaging (69%), which is done via cone-beam CT (CBCT) in five or portal imaging in four institutions. The latter is accompanied by CBCT once a week in three institutions. Four centers use daily CBCT in the first 3–5 days and schedule the following IGRT strategy according to the results.

### Systemic chemotherapy

All institutions indicated the use of doublet regimens concurrent to RT including mitomycin (100%) and 5‑fluorouracil as continuous infusion (46%) or its prodrug capecitabine (54%). Eleven institutions made specific dose recommendations listed in Table 5. Systemic therapy is administered in the radiation oncology department in the majority of centers (62%).

**Table 5:** Chemotherapy regimens

	MMC	5‑FU	Cap
*n* = 5	2 doses of 10 mg/sqm^1^	–	825 m/sqm bid^4^
*n* = 3	2 doses of 10 mg/sqm^1^	1000 mg/sqm, 4 cons. days^3^	–
*n* = 1	2 doses of 10 mg/sqm^1^	1000 mg/sqm, 5 cons. days^3^	–
*n* = 1	1 dose of 12 mg/sqm^2^	1000 mg/sqm, 4 cons. days^3^	–
*n* = 1	1 dose of 12 mg/sqm^2^	750 mg/sqm, 5 cons. days^3^	–

*n* number of centers, *MMC* mitomycin C, *5‑FU* 5-fluorouracil, *Cap* capecitabine, *sqm* square meter, *bid* two times per day, *cons.* Consecutive

^1^applied at one day each in week 1 and 5

^2^applied at day one in week 1 only

^3^applied as continuous infusion in week 1 and 5

^4^applied only during days of radiation treatment

### Supportive treatment

The treatment procedure is performed on an outpatient basis in the majority of centers (69%), while 31% indicated to constantly treat patients on their ward. Specific supportive treatments beyond skin care are offered as nutritional advice in 38%, psycho-oncological support in 23%, and prevention of vaginal stenosis in 54% of the centers.

### Follow-up

All institutions offer regular follow-up visits, including specific visits in the RO department in 10 centers (77%). Follow-up is offered regularly for 5 years in 77% and for 8–10 years in 15%. Visits are usually scheduled every 3 months for the first year, every 3–6 months for the second year, and every 6–12 months thereafter. Included examinations are listed in Table 6. In case of incomplete clinical remission, 54% of the centers perform biopsies at 3 months and 46% of the centers at 6 months.

**Table 6:** Examinations during follow-up

Examinations	*N*	%
DRE	11	85
Pelvic MRI	10	77
Procto-/rectoscopy	8	62
Abdominal CT	8	62
Chest CT	7	54
Colonoscopy	2	15
Endorectal ultrasound	2	15
Inguinal ultrasound	2	15
PET-CT	1	8
Abdominal ultrasound	1	8
Tumor marker (SCC)	1	8

*n* number of centers, *%* percentage of centers, *DRE* digital rectal examination, *MRI* magnetic resonance imaging, *CT* computed tomography, *PET-CT* positron emission computed tomography, *SCC* squamous cell carcinoma antigen

## Discussion

With a response rate of 93%, our survey represents a complete and valid image of the practice in Austria, clearly outmatching values reached in other countries. For example, a similar German survey recently reported only a 28% response rate [3]. We evaluated areas of agreement and disagreement between the centers and compared the general approach with the recommendations of major international guidelines, namely NCCN [1] and ESMO-ESSO-ESTRO (ESMO) [2] in their latest version.

### Work-up

There is considerable agreement on most work-up and staging issues between the centers and with regard to current guidelines [1, 2]. Areas of disagreement mainly include specific additional staging procedures. For example, 54% of the centers indicated the use of a complete colonoscopy, although not recommended according to either guideline [1, 2]. The same is true for endorectal ultrasound (39%), which is not routinely required according to NCCN [1]. In contrast, PET-CT is used for staging in only a minority (38%), although recommended (if available) by NCCN [1] and ESMO [2] for possible advantages in staging accuracy and target volume delineation [4–6]. Only 15% of the Austrian centers indicated routine HIV testing, which is even lower than the 27% rate reported in the recent German survey [3]. This might be due to the fact that according to Austrian regulations, HIV testing requires a special informed consent form. Only 54% of the centers indicated routine HPV testing of biopsies, although evidence for HPV positivity as a positive predictive marker for outcome is growing [7, 8]. This might be because only one center indicated altering the treatment concept based on HPV status or because current guidelines [1, 2] do not recommend routine HPV testing nor guidance of treatment decisions by HPV status to date. Good accordance with major guidelines was also found regarding the recommendations for gynecological examinations and counseling for potential infertility. Interestingly, only 15% of the centers perform biopsies in case of suspicious inguinal LN, although recommended by both guidelines [1, 2], while inguinal ultrasound is used by 23%, although not recommended in either one. This practice might be explained by the absence of clear evidence regarding improved staging accuracy with biopsies, or by the fact that most centers indicated using dose escalation in suspicious LN.

### Simulation/treatment planning/treatment procedure

Detailed recommendations specifically dealing with treatment planning are given to some extent in the guidelines. Supine treatment position is preferred by both [1, 2], while prone position is advocated only based on individual decisions. The latter is also true for the use of bolus material. Intravenous and/or oral contrast-enhanced CT for treatment planning and marking of the lower tumor edge is recommended only by NCCN [1]. Austrian centers are mainly in line with these recommendations. However, only a minority indicated the use of intravenous and/or oral contrast-enhanced planning CT (38%). Because of superior dose distributions and reduced toxicity [9–17], both guidelines [1, 2] clearly recommend intensity-modulated techniques, which has been adopted by all Austrian centers. Similarly, daily kV image guidance is advocated by NCCN [1], which is also preferred by the majority of Austrian centers.

### Target volume definition/dose prescription

While NCCN [1] provides specific dose ranges for different disease stages and detailed recommendations regarding the target volume, ESMO [2] includes only very general advice regarding this issue. Therefore, guideline adherence is discussed mainly with regard to NCCN [1].

Regarding total dose to the PT, NCCN [1] recommends a boost beyond the doses prescribed for elective nodal areas of 5.4–14.4 Gy depending on T‑stage, resulting in total doses of 50.4–59.4 Gy (shrinking-field) or 50.4–54 Gy (SIB techniques). This recommendation has been adopted by nearly all Austrian centers, although most prefer sequential boosting. Most centers further indicated prescribing higher total doses in locally advanced primaries (median total 54.7 Gy for T1–2 and 59.4 Gy for T3–4 tumors). While these doses are still in line with the current guideline recommendations [1, 2], an even more personalized approach with further dose de-escalation in early stages and further dose escalation in advanced stages is currently being evaluated in several prospective trials (e.g., by the PLATO platform, ISRCTN88455282, or the DECREASE trial, NCT04166318).

Moreover, the vast majority (85%) indicated use of a boost to enlarged LN (median dose 54 Gy, range 50–60 Gy), mainly restricted to the LN itself rather than the affected region. While the preferred technique of boosting only the involved node is in line with the NCCN [1], the median reported boost dose is equivalent to the dose recommended only for nodes > 3 cm [1]. Interestingly, only one center indicated different doses depending on LN size, although recommended by NCCN [1].

We asked detailed questions regarding the covered elective nodal areas for cN0 and cN1 situations. Generally, high concordance rates between the centers exist regarding both scenarios. Most centers (85%) regularly include the bilateral inguinal, iliac external, iliac internal, mesorectal, presacral, and obturator nodes with an even slightly increased concordance rate for node-positive patients (92%). This pattern does exactly match the NCCN recommendations [1]. The only matter of debate seems to be the common iliac node area. While in cN0 patients, 46% indicated the inclusion of this area, this rate increased even further to 62% in cN1 patients. This finding seems somewhat surprising, as neither NCCN [1] nor ESMO [2] recommends the inclusion of this area in either situation, although increasing evidence suggests that its inclusion might be justified in high-risk situations [18–20].

Prescription doses to elective nodal volumes distinctly differ between major trials [10, 13, 14, 21], although all prescribed 30.6–45 Gy. Consequently, NCCN [1] generally recommends this dose range, while ESMO [2] just recommends including “any sites of likely nodal involvement” with no specific dose recommendation. All centers indicated doses to elective nodal regions within the recommended dose ranges or above. The reported median doses are higher for cN+ cases (compared to cN0), suggesting the assumption of controlling a larger amount of subclinical disease with slightly increased doses in cN+ patients. The median reported doses are above the recommended range, especially for cN+ patients (50–50.4 Gy) and to a lesser extent for cN0 patients, although this has not been evaluated or proven by randomized trials.

Regarding dose constraints for OAR, only NCCN [1] but not ESMO [2] provides specific recommendations. The answers given by the Austrian centers showed a large diversity of generally considered OARs and large variations in accepted doses. Contouring OARs and using specific dose constraints for AC might be a field of further improvement.

### Systemic chemotherapy

Very strong agreement among Austrian centers and accordance with guidelines exists regarding the chemotherapy regimens for simultaneous CRT. All institutions indicated the use of a doublet including mitomycin (MMC) and 5‑FU or capecitabine, which both showed benefits in two large trials [13, 21, 22]. Capecitabine is preferred over infusional 5‑FU by a slight majority, which may either reflect its easier application or be because retrospective data suggests lower hematological toxicity compared to infusional 5‑FU in patients receiving IMRT [23].

### Supportive care

Most centers perform CRT mainly on an outpatient basis, although 31% indicated regularly treating their patients on their wards. This may reflect the assumption of a need for intensified care for treatment side effects or the sometimes large travel distances due to the “centralized” structure of radiation oncology care in Austria as well as the country’s geography.

Clear recommendations regarding supportive care for specific side effects are rarely given by major guidelines, although NCCN provides detailed general recommendations in a specific guideline (NCCN principles of survivorship [24]). To cover this complex subject, we asked two questions referring to generally recommended issues [2, 24] and one to a specific late toxicity. Interestingly, only a minority of centers regularly offers nutritional advice (38%) or psycho-oncological support (23%), although both issues affect the majority of surviving patients after pelvic RT [25, 26]. In contrast, 54% advise female patients to use vaginal dilatators to prevent stenosis, which represents a high level of awareness compared to other reports on sexual dysfunction after pelvic RT [26].

### Follow-up

Recommendations regarding follow-up investigations after chemoradiation for AC distinguish between response evaluation and follow-up in case of complete remission. The main issue in response evaluation is the timepoint at which to consider histological confirmation of clinically persistent disease for potential salvage surgery. Austrian centers are divided roughly equally between a 3-month and a 6-month interval, although both guidelines [1, 2] clearly favor the latter timepoint based on the data from ACT II [27].

Regarding follow-up after complete remission, major guidelines favor DRE [1, 2] supported by inguinal node palpation [1, 2] and anoscopy [1] every 3–6 months for 5 years [1], while cross-sectional imaging is recommended at larger intervals and/or only in advanced disease [1]. Accordingly, all Austrian institutions offer regular follow-up using the recommended methods at 3‑month intervals for the first 1–2 years, which are increased to 6–12 months over time. However, those visits usually include cross-sectional imaging (mainly pelvic MRI and chest/abdominal CT) at equal intervals.

## Conclusion

In summary, we found high rates of agreement between the centers and concordance with the recommendations of international guidelines, at least covering the main issues of work-up, treatment, and follow-up for CRT of AC. Only PET-CT, routine HIV testing, and biopsy of suspicious LN seem to be less frequently used than recommended. While high agreement and concordance to guidelines are found in general with regard to modern radiation techniques and elective nodal target volumes, large controversy exists regarding inclusion of the common iliac nodes. Prescription doses vary to some extent, but are generally in line with the recommendations, although sometimes at or above the recommended upper dose range. In contrast, no agreement on delineation of OARs or dose constraints exists, which raises a possible need for standardization. The same is true for supportive care during/after CRT, which is underrepresented in major guidelines although this is assumed to be an integral part of the treatment [28]. Follow-up is performed even more intensively than recommended. Considerable disagreement regarding treatment of early AC exists, indicating a need for further research.

## Supplementary Information

Full text with extended results and discussion especially regarding the treatment of T1N0 anal cancer

Questions

## Abbreviations

5‑FU: 5 Fluorouracil
AC: Anal cancer
CBCT: Cone-beam computed tomography
CRT: Chemoradiation
CT: Computed tomography
CTV: Clinical target volume
DRE: Digital rectal examination
ESMO: European society for medical oncology
GTV: Gross tumor volume
HIV: Human immunodeficiency virus
IGRT: Image-guided radiation therapy
IMRT: Intensity modulated radiation therapy
LN: Lymph node
MMC: Mitomycin C
MRI: Magnetic resonance imaging
NCCN: National comprehensive cancer network
OAR: Organs at risk
ÖGRO: *Östereichische Gesellschaft für Radio-Onkologie*
ÖGRO-GIT: *Östereichische Gesellschaft für Radio-Onkologie – Gastrointestinealer Tumoren*
PET-CT: Positron-emission tomography computed tomography
PT: Primary tumor
PTV: Planning target volume
RO: Radiation oncology
RT: Radiation treatment/radiotherapy
SIB: Simultaneous integrated Boost
SOP: Standard operating procedures
TNM: Tumor Node Metastases
VMAT: Volumetric intensity-modulated radiation therapy
