# Supplementary material for: Practice of radiation therapy for anal cancer in Austria—a survey on behalf of the Austrian radiation oncology society gastrointestinal tumor group (ÖGRO-GIT)
Source: Strahlenther Onkol. 2021 Sep 30;197(11):953–61. doi: 10.1007/s00066-021-01842-w (PMC8547205; doi:10.1007/s00066-021-01842-w)
Supplement: Supplementary file 1 — Full text with extended results and discussion especially regarding the treatment of T1N0 anal cancer [file 66_2021_1842_MOESM1_ESM.docx]

**Practice of Radiation Therapy for Anal Cancer in Austria – A Survey on behalf of the Austrian Radiation Oncology Society Gastrointestinal Tumor Group (ÖGRO-GIT)**

Gerum S^1^, Iglseder W^1^, Schmid R², Peterka K³, Knocke-Abulesz TH⁴, Harl P⁵, Schwaiger S⁶, Reiter I⁷, Salinger J⁸, Venhoda C⁹, Kurzweil G¹⁰, Poetscher M¹¹, Jaeger R¹², Celedin B¹³, Clemens P¹⁴, Roeder F^1^

^1^ Universitätsklinik für Radiotherapie und Radio-Onkologie, Landeskrankenhaus Salzburg, Uniklinikum der Paracelsus Medizinischen Universität, Müllner Hauptstrasse 48, 5020 Salzburg, Austria, s.gerum@salk.at

² Universitätsklinik für Radioonkologie, Medizinische Universität Wien, Universitätsklinikum AKH Wien, Comprehensive Cancer Center Vienna, Währinger Gürtel 18-20, 1090 Wien, Austria, r.schmid@meduniwien.ac.at

³ Institut für Radioonkologie, Kaiser-Franz-Josef-Spital / SMZ Süd-Klinik Favoriten, Kundratstrasse 3, 1100 Wien, Austria, karin.peterka@gesundheitsverbund.at

⁴ Sonderabteilung Strahlentherapie, Wiener Gesundheitsverbund Klinik Hietzing, Wolkersbergenstrasse 1, 1130 Wien, Austria, thomas-hendrik.knocke-Abulesz@gesundheitsverbund.at

⁵ Institut für Radioonkologie, SMZ – Ost Donauspital der Stadt Wien, Langobardenstrasse 122, 1220 Wien, Austria, phillipp.harl@gesundheitsverbund.at

⁶ Institut für Radioonkologie, Klinik Ottakring, Wilhelminenspital der Stadt Wien, Montleartstrasse 37, 1160 Wien, Austria, [susanne.schwaiger@gesundheitsverbund.at](mailto:susanne.schwaiger@gesundheitsverbund.at)

⁷ Institut für Radioonkologie und Strahlentherapie, Landesklinikum Wiener Neustadt, Corvinusring 3-5, 2700 Wiener Neustadt, Austria, [irene.reiter@wienerneustadt.lknoe.at](mailto:irene.reiter@wienerneustadt.lknoe.at)

⁸ Klinische Abteilung für Strahlentherapie- Radioonkologie, Universitätsklinikum Krems, Karl Landsteiner Privatuniversität für Gesundheitswissenschaften, Mitterweg 10, 3500 Krems an der Donau, Austria, johanna.salinger@krems.lknoe.at

⁹ Klinik für Radioonkologie, Klinikum der Barmherzigen Schwestern, Ordensklinikum Linz, Seilerstätte 4, 4010 Linz, Austria, clemens.venhoda@ordensklinikum.at

¹⁰ Klinik für Radioonkologie/Strahlentherapie, Salzkammergutklinikum Vöcklabruck, Dr.-Wilhelm-Boch-Strasse 1, 4840 Vöcklabruck, Austria, gebhard.kurzweil@ooeg.at

¹¹ Universitätsklinik für Strahlentherapie -Radioonkologie, Comprehensive Cancer Center Graz, Medizinische Universität Graz, Auenbruggerplatz 32, 8036 Graz, Austria, michaela.poetscher@klinikum-graz.at

¹² Universitätsklinik für Strahlentherapie -Radioonkologie, Medizinische Universität Innsbruck, Anichstrasse 35, 6020 Innsbruck, Austria, robert.jaeger@tirol-kliniken.at

¹³ Institut für Strahlentherapie/Radioonkologie, Klinikum Klagenfurt am Wörthersee, Feschnigstrasse 11, 9020 Klagenfurt, Austria, [bettina.celedin@kabeg.a](mailto:bettina.celedin@kabeg.a)t

¹⁴ Institut für Radioonkologie und Strahlentherapie, Landeskrankenhaus Feldkirch, Carinagasse 47, 6807 Feldkirch, Austria, [Patrick.Clemens@lkhf.at](mailto:Patrick.Clemens@lkhf.at)

Corresponding Author: Dr. Sabine Gerum

Department of Radiotherapy and Radiation Oncology

Paracelsus Medical University

Landeskrankenhaus

Müllner Hauptstrasse 48

5022 Salzburg

Austria

[s.gerum@salk.at](mailto:s.gerum@salk.at)

Phone: +43 5 7255 57031

**Abstract**

**Purpose**

Chemoradiation is the mainstay of treatment for locally confined anal cancer. While the general indication is widely accepted, international consensus for adequate staging procedures, radiation technique, dose and fractionation, target volume definition, supportive care, follow-up and treatment of early lesions is less uniform. We therefore conducted a nationwide patterns-of-care survey in Austria to evaluate areas of disagreement and to identify possible targets for further standardization and research.

**Methods**

We developed an anonymous questionnaire comprising 38 questions for the above mentioned issues. The survey was sent to all 14 Austrian radiation oncology departments. Results were analyzed descriptively and compared to two major international guidelines (NCCN and ESMO) in their latest version.

**Results**

We received 13 answers (response rate of 93%). Median number of treated patients per year and institution was 14. Work-up generally included DRE, endoscopy and cross-sectional imaging of chest, abdomen (mainly CT) and pelvis (mainly MRI). PET-CT was used by 38%. Screening for HIV and biopsies of suspicious lymph nodes (LN) (15% each) were infrequently used. All centers use IMRT with some kind of (mainly daily) IGRT. Median doses to the primary tumor were 54.7 Gy (50.4-59.4) for T1-2 and 59.4 Gy (55-64.4) for T3-4 lesions. Boosts are applied mainly sequentially. Doses to elective nodal areas varied from 30.6 to 60 Gy depending on whether the patient was cN0 or cN+, but most centers prescribe 45-50.4 Gy to all elective nodal areas. Suspicious nodes usually receive a boost independent of their size to a median dose of 54 Gy (50-60 Gy). Target delineation of elective nodal areas seems generally uniform with inclusion of the common iliac nodes as the only area of disagreement. No agreement was found for OAR delineation and dose constraints. Concurrent chemotherapy was Mitomycin and 5-FU/Capecitabine in all centers with 54% favoring capecitabine. Supportive care like nutritional counseling or psycho-oncological care was infrequently offered. Intensive follow-up was performed by all institutions for at least 5 years. Treatment of T1N0 showed considerable disagreement (46% surgery, 31% radiation alone, 23% chemoradiation). Median dose to primary tumor was 57.2 Gy (50-60 Gy) and 2/7 centers favouring (C)RT indicated to treat no elective nodal areas in this situation.

**Conclusion**

We found a high rate of agreement between the centers and concordance with the recommendations of major guidelines at least for the main issues of work-up, treatment and follow-up. PET-CT, routine HIV testing and biopsies of suspicious LN seem underrepresented. The largest controversy regarding elective nodal target volumes concerns inclusion of the common iliac nodes. Doses to primary tumor, suspicious LN and elective nodal areas vary to some extent and are mainly in line with the recommendations, although sometimes above. OAR delineation, dose constraints, supportive care and treatment of early anal cancer are highly variable and represent areas for further standardization and research.

**Background**

Definitive Chemoradiation (CRT) has gradually emerged as the standard of care for curative intent treatment of locoregionally confined anal cancer since the encouraging first results demonstrated by Nigro et al. in the early 70s [1]. Its preference to surgery is mainly based on its high potential for sphincter-preservation without compromising oncological outcome [2,3]. Except for T1N0 anal cancer, simultaneous CRT is clearly favorably compared to radiotherapy alone based on randomized data [4]. While the general indication for CRT in locally advanced, non-metastatic anal cancer is widely accepted, international consensus for some issues like adequate staging procedures, radiation technique, dose and fractionation, target volume definition, supportive care, patient counseling in special situations and treatment of early lesions is heterogeneous. Moreover, imaging and radiation techniques as well as treatment approaches regarding preexisting comorbidities or side effects have clearly emerged over the past decades, indicating the need for continuous adaption of treatment and care strategies. For instance, a recent multi-institutional phase II trial (RTOG 0529) demonstrated a lower rate of acute toxicities with IMRT using rather low total doses compared to prior randomized trials [5]. Since not all pending questions can (or likely will) be addressed in randomized or prospective trials, evidence of lower levels including expert opinions must be taken into account for the development of guidelines, which consequently may differ distinctly. Moreover, knowledge of the actual pattern of care is crucial for the development of future prospective studies. Surveys generally offer an easy possibility to analyze patterns of care of a specific region, however their value is often limited by low rates of response. In this regard, regions or nations using more centralized care systems with a limited number of centers treating the majority of patients with a certain disease may offer some advantages. In Austria, access to radiation therapy is currently limited to 14 institutions (plus one particle center), which can be addressed easily via the Austrian Society of Radiation Oncology (ÖGRO), specifically its gastrointestinal working group (ÖGRO-GIT). We therefore conducted a survey regarding the radiation treatment of locoregionally confined anal cancer in order to compare actual patterns of care in Austria with the recommendations of major international guidelines, to identify possible areas of controversies and hence, to guide the development of future studies and Austrian guidelines.

**Methods**

All 14 Austrian radiation oncology institutions were invited via email to take part in the survey, which was restricted to questions regarding the regular treatment of squamous-cell, locoregionaly confined, non-metastatic anal cancer in the respective institution. The survey included 38 questions in total with either a predefined choice of answers or for written description of the centers approach (see supplementary material). Description of an individual approach not covered by predefined answers was possible for any issue. Five questions covered general issues of the radiation department, nine covered indication and staging procedures, and eighteen radiation treatment planning, target volume definition, dose and fractionation, dose constraints, chemotherapy or radiation delivery. The remaining five questions covered supportive care during treatment and follow-up. Because of the known controversies in treating early stage anal cancer (T1N0), we added a clinical example and asked for the centers opinion regarding six issues of the optimal treatment approach and delivery. The survey was made available on an online platform allowing anonymous participation. All centers were guided to strictly send only one answer per center and to indicate via email if they had participated or not. Numerical variables were analyzed descriptively with IBM SPSS statistics Version 24 and described as median and range. Free-text answers were analyzed separately. Results were descriptively compared to the actual versions of international multinationally used multidisciplinary guidelines from the US [2] and Europe [3] in the absence of a multidisciplinary Austrian guideline. A comparison with other national guidelines (for example German, French or UK guidelines) was intentionally not performed.

**Results**

**Response rate and general information**

Of 14 radiation therapy centers in Austria 13 centers responded, resulting in a response rate of 93%, of which 5 were university-bound centers. Most institutions (n=9, 69%) have 1-3 linear accelerators in use, three (27%) are equipped with 4-6 and only one center with more than 6 accelerators. The median number of patients with primary, non-metastatic anal cancer treated with curative intent (chemo)-radiation per year and per institution was 14 (7-35). Written Standard operating procedures (SOP) for the radio-oncological treatment procedure of anal cancer are available in 46% of the institutions, while no center reported clinical trials actually recruiting anal cancer patients.

**Work-up**

All responses were deemed eligible for analysis. Procedures routinely used for diagnostic work-up are listed in table 1. Clinical examination is performed in all centers although only 85% literally reported digital rectal examination (DRE). All centers perform some form of endoscopy (either procto-rectoscopy or colonoscopy) with histological confirmation and pelvic MRI for locoregional staging. Endo-rectal ultrasound (39%), ultrasound of inguinal LN (23%), or biopsies of suspicious inguinal LN (15%) are infrequently performed as additional locoregional staging modalities. For ruling out of distant metastases, all centers perform some form of chest (either X-rays, CT or PET-CT) and abdominal (either ultrasound, CT or PET-CT) imaging, however PET-CT is routinely used only in a minority (38%) of the centers. Routine HIV-testing is done only in 2 centers (15%) and does not change the general treatment principles. Routine HPV-testing of the pathological specimen of the primary tumor is performed in 54%, although altering the treatment regime only in one center. Female patients generally receive a specific gynecological clinical examination in 62% of the centers and only in case of suspected vaginal involvement in further 15% of the centers. Male patients wishing to preserve fertility or female patients with child-bearing potential are routinely counselled with regard to fertility-protection or cryopreservation in 77% of the centers. Multidisciplinary discussion of the case prior to its initiation is routinely done in 85% of the centers.

Table 1: Examinations for work-up

| **Examination** | **N** | **%** |
| --- | --- | --- |
|  |  |  |
| pelvic MRI | 13 | 100 |
| procto-rectoscopy | 12 | 92 |
| DRE | 11 | 85 |
| abdominal CT | 11 | 85 |
| gyn. examination* | 10 | 77 |
| counseling (fertility protection) | 10 | 77 |
| chest CT | 9 | 69 |
| Colonoscopy | 7 | 54 |
| HPV status (biopsy) | 7 | 54 |
| endorectal ultrasound | 5 | 39 |
| PET-CT | 5 | 39 |
| inguinal ultrasound | 3 | 23 |
| biopsy of suspicious inguinal nodes | 2 | 15 |
| HIV status | 2 | 15 |
| chest X-ray | 1 | 8 |
| abdominal ultrasound | 1 | 8 |
| tumor marker (SCC) | 1 | 8 |

n: number of centers, %: percentage of centers, MRI: magnetic resonance imaging, DRE: digital rectal examination, CT: computed tomography, gyn.: gynecological, HPV: human papilloma virus, PET-CT: positron-emission computed tomography, HIV: human immunodeficiency virus, SCC: squamous cell carcinoma antigen

**Treatment planning and delivery**

The questionnaire included detailed questions regarding target volume definition, doses and fractionation and chemotherapy regimens. Because early stage anal cancer (T1N0) may inherit distinct differences regarding the general treatment approach and delivery, we excluded early stage anal cancer from those queries and specifically addressed its treatment in a case example (see own paragraph). Therefore, the following results correspond to locoregionally confined anal cancer (T2-4 N0-1 M0).

**Simulation/Treatment planning**

Treatment-planning CT is done in prone position in only one institution, while the remaining centers (92%) prefer treatment planning in supine position. Oral or intravenous contrast agents are used in 8% and 31% of the centers, respectively. Patients are required to have a full bladder in all institutions (100%), and 8 centers (62%) also advise an empty rectum. The lower edge of the primary tumor (if visible) or the anal verge is specifically marked with radio-opaque material in 77%, while only 23% of the centers mark the vagina in female patients. Four institutions (31%) indicated the use of bolus material in case of a prolapsed primary (two in general; two only in case of insufficient dose coverage).

**Target volume definition and Prescription dose**

As target volumes might depend on clinical (in particular nodal) stage, we asked the participants to indicate their choice of elective lymph nodes regions for a CTV in cN0 and cN+ situations, respectively. We further asked to specify the total and single doses for each electively treated region.

For node negative cases, all centers include the bilateral inguinal and internal iliac nodes into the CTV, most centers (85-92%) also include the external iliac, mesorectal, presacral and obturator nodes, but only a minority (46%) would include the common iliac nodes (table 2). Total doses ranged between 30.6 Gy and 54 Gy in conventional fractionation (single dose 1.65-2 Gy), with the majority of centers (n=7) indicating total doses of 45-50.4 Gy for all elective nodal regions with fractional doses of 1.8 Gy.

Table 2: elective nodal volumes and prescription doses in cN0 patients

|  |  |  |  |  |
| --- | --- | --- | --- | --- |
| **elective nodal region** | **n** | **%** | **total dose** | **single dose** |
|  |  |  |  |  |
| bilateral inguinal nodes | 13 | 100 | 45 (30.6-50.4) | 1.8 (1.65-2) |
| bilateral internal iliac nodes | 13 | 100 | 46 (30.6-50.4) | 1.8 (1.65-2) |
| bilateral external iliac nodes | 11 | 85 | 47.3 (39.6-50.4) | 1.8 (1.65-2) |
| bilateral common iliac nodes | 6 | 46 | 47.9 (39.6-50.4) | 1.8 (1.65-2) |
| mesorectal nodes | 12 | 92 | 49.5 (30.6-54) | 1.8 (1.7-2) |
| presacral nodes | 12 | 92 | 46.4 (30.6-50.4) | 1.8 (1.7-2) |
| bilateral obturator nodes | 12 | 92 | 47.3 (39.6-50.4) | 1.8 (1.6-2) |

cN0: clinically node negative case, n: number of centers who would include the region, %: percentage of centers who would include the region, total dose: median total dose for this region (range of doses for this region), single dose: median single dose for this region (range of single doses for this region), if a center specified a dose range, the mean of the dose range was used for calculation of the median values

For node positive cases, all centers include the bilateral inguinal, internal iliac, external iliac, mesorectal and obturator LN into the CTV. All but one (92%) would also include the presacral nodes, and 62% the common iliac nodes (table 3). Total doses ranged from 30.6 Gy to 60 Gy in conventional fractionation (single doses 1.65-2 Gy), although the median total doses were slightly higher for all elective nodal regions compared to the cN0 patients. Again the majority of centers (n=8) indicated total doses of 45-50.4 Gy for all elective nodal regions in 1.8 Gy single doses.

Table 3: elective nodal volumes and prescription doses in cN+ patients

|  |  |  |  |  |
| --- | --- | --- | --- | --- |
| **elective nodal region** | **n** | **%*** | **total dose** | **single dose** |
|  |  |  |  |  |
| bilateral inguinal nodes | 13 | 100 | 50 (30.6-60) | 1.8 (1.7-2) |
| bilateral internal iliac nodes | 13 | 100 | 50.4 (30.6-50.4) | 1.8 (1.7-2) |
| bilateral external iliac nodes | 13 | 100 | 50.4 (30.6-50.4) | 1.8 (1.65-2) |
| bilateral common iliac nodes | 8 | 62 | 50.2 (45-50.4) | 1.8 (1.65-2) |
| mesorectal nodes | 12 | 92 | 50.4 (30.6-54) | 1.8 (1.7-2) |
| presacral nodes | 13 | 100 | 50.2 (30.6-54) | 1.8 (1.7-2) |
| bilateral obturator nodes | 13 | 100 | 50.4 (30.6-50.4) | 1.8 (1.7-2) |

cN+: clinically node positive case, n: number of centers who would include the region, %: percentage of centers who would include the region, total dose: median total dose for this region (range of doses for this region), single dose: median single dose for this region (range of single doses for this region), if a center specified a dose range, the mean of the dose range was used for calculation of the median values

Most centers (77%) increase the total dose (boost) in suspicious nodes up to a median dose of 54 Gy (range 50-60Gy), mainly independent of LN size. One center increases the total dose in the entire affected lymph node region, one center does not change the dose and one center didn´t answer the corresponding question. All institutions used conventional or slightly accelerated fractionation for boosting the lymph node or lymph node area (single doses 1.8-2.2 Gy).

Dose escalation in the primary tumor region is generally performed by 92% (n=12), either as sequential (54%) or simultaneous integrated (15%) external beam photon boost, as electron boost (15%) or via brachytherapy (15%). Prescription of a boost, total dose and fractionation seem to depend on tumor size. For small tumors (cT1-2), only 85% generally prescribe a boost, while all prescribe a boost for larger primaries (cT3-4). Ten centers (77%) explicitely indicated to increase the total dose in cT3-4 tumors compared to cT1-2 tumors. For cT1-2 primaries, a median total dose of 54.7 Gy (50.4-59.4 Gy) in conventional or slightly accelerated fractionation (single doses 1.8-2.2 Gy) was prescribed by the centers using external beam boosts, while larger tumors (cT3-4) would receive a median total dose of 59.4 Gy (55-64.4 Gy) in single doses of 1.8-2.3 Gy.

**Treatment Procedure**

All centers indicated to perform volumetric intensity-modulated radiation therapy (VMAT), no one uses step-and-shoot IMRT, 3D-conformal RT or 2D techniques. Participants were asked to provide information on generally outlined organs at risk (OAR) and if they use specific dose constraints or just try to keep the dose reasonably low during the planning process (see table 4). The given dose constraints varied widely even for a single organ at risk (data not shown).

Table 4: outlined organs at risk (OAR) and use of specific dose constraints

|  | **Outlined** | | **specific contraints** | |
| --- | --- | --- | --- | --- |
| **organ at risk** | **n** | **%** | **n** | **%** |
|  |  |  |  |  |
| bladder | 13 | 100 | 10 | 77 |
| femoral head | 10 | 77 | 8 | 61 |
| small bowel | 9 | 69 | 5 | 38 |
| bowel bag | 8 | 61 | 6 | 46 |
| colon | 5 | 38 | 1 | 8 |
| external genitalia | 5 | 38 | 3 | 23 |
| cauda equina | 2 | 15 | 2 | 15 |
| pelvic bone | 1 | 8 | 0 | 0 |

n: number of centers, %: percentage of centers

Some form of image-guided radiation therapy (IGRT) is performed by all centers (100%), but technique and timing varies. Most departments indicated daily imaging (n=9, 69%), which is done via cone-beam CT (cbct) in five or portal imaging in four institutions. The latter is accompanied by cbct once a week in three institutions. Four centers use daily cbct in the first 3-5 days and schedule the following IGRT strategy according to the results.

**Systemic chemotherapy**

All institutions indicated the use of doublet regimens concurrent to radiation treatment including mitomycin C (100%) and 5-fluorouracil as continuous infusion (46%) or its prodrug capecitabine (54%). Eleven institutions made specific dose recommendations listed in table 5.

Table 5: chemotherapy regimens

|  | **MMC** | **5-FU** | **Cap** |
| --- | --- | --- | --- |
|  |  |  |  |
| n=5 | 2 doses of 10 mg/sqm^1^ |  | 825 m/sqm bid^4^ |
| n=3 | 2 doses of 10 mg/sqm^1^ | 1000 mg/sqm, 4 cons. days^3^ |  |
| n=1 | 2 doses of 10 mg/sqm^1^ | 1000 mg/sqm, 5 cons. days^3^ |  |
| n=1 | 1 dose of 12 mg/sqm^2^ | 1000 mg/sqm, 4 cons. days^3^ |  |
| n=1 | 1 dose of 12 mg/sqm^2^ | 750 mg/sqm, 5 cons. days^3^ |  |

n: number of centers, MMC: mitomycin C, 5-FU: 5-fluorouracil, Cap: capecitabine, mg: milligramm, sqm: square meter, bid: two times per day, cons.: consecutive, 1: applied at one day each in week 1 and 5, 2: applied at day one in week 1 only, 3: applied as contineous infusion in week 1 and 5, 4: applied only during days of radiation treatment

Systemic therapy is administered in the radiation oncology department in 62% and in the medical oncology department in 23%, while two centers did not answer this question.

**Supportive treatment**

Treatment procedure is performed on an out-patient basis in the majority of centers (n=9, 69%) with possible hospitalisation during chemotherapy or in case of major side effects in seven institutions (54%), while in 31%, centers indicated to constantly treat patients on their ward. Specific supportive treatments beyond skin care are offered as nutritional advice in 38%, psycho-oncological support in 23% and prevention of vaginal stenosis in 54% of the centers. One center regularly offers pelvic floor training to their patients.

**Follow up**

All institutions offer regular follow-up visits to their patients. Specific follow-up visits in the radiation oncology department are offered in 10 centers (77%), which are accompanied by follow-up visits in the surgical and/or medical oncological department in 6 centers. Exclusive follow-up in other departments is advocated by three centers (surgical department:1, medical oncological department:1, both:1). Follow-up is offered regularly for 5 years in 77% of the centers and for 8-10 years in 15%, while one institution did not answer this question. Follow-up visits are scheduled every three months for the first year in all institutions that answered this question (n=12). This interval is maintained in 7 (58%), increased to 6 months in 4 (33%) and to 12 months in one center for the second year. From the beginning of the third year, 67 % perform follow-up visits every 6 months and 33% every 12 months. Examinations included into the follow-up visits are listed in table 6.

Table 6: Examinations during follow-up

| **Examinations** | **N** | **%** |
| --- | --- | --- |
|  |  |  |
| DRE | 11 | 85 |
| pelvic MRI | 10 | 77 |
| procto-Rectoscopy | 8 | 62 |
| abdominal CT | 8 | 62 |
| chest CT | 7 | 54 |
| colonoscopy | 2 | 15 |
| endorectal ultrasound | 2 | 15 |
| inguinal ultrasound | 2 | 15 |
| PET-CT | 1 | 8 |
| abdominal ultrasound | 1 | 8 |
| tumormarker (SCC) | 1 | 8 |

n: number of centers, %, percentage of centers, DRE: digital rectal examination, MRI: magnetic resonance imaging, CT: computed tomography, PET-CT: positron emission computed tomography, SCC: squamous cell carcinoma antigen

In case of incomplete clinical remission, 54% of the centers would perform biopsies to rule out residual disease at three months from the end of radiation therapy and 46% of the centers at six months.

**Case scenario – early stage anal cancer – How to treat?**

We asked the participating institutions how they specifically would treat a 50 year old female patient without significant comorbidities, who presents with histologically confirmed early anal cancer staged as cT1 cN0 cM0 G1, located in the transition zone from anal mucosa to anal skin.

The generally preferred treatment option was definitive (chemo)radiation in 7 (54%) and local excision in 6 (46%) centers. Of the 7 institutions which preferred non-surgical treatment, 4 would use radiation therapy only, and 3 combined CRT. All 7 centers would include the primary tumor region, with some form of elective nodal irradiation advocated by 5 centers (table 7). Prescription dose for the primary tumor varied between 50.4 and 60 Gy (median 57.2 Gy) and between 30.6 and 50 Gy for different elective nodal regions (table 7). Most centers used conventional fractionation with single doses of 1.8-2.2 Gy (median 1.9 Gy) for the primary tumor and 1.65-2 Gy for the elective nodal regions. Of the three centers favouring combined CRT, one would use Mitomycin C/Capecitabine and two would prefer Mitomycin C/5-FU.

| Table 7: elective nodal target volumes and prescription doses for early stage anal cancer (n=7)   \| **cT1N0 G1** \|  \|  \|  \|  \| \| --- \| --- \| --- \| --- \| --- \| \| **elective nodal region** \| **n** \| **%** \| **total dose** \| **single dose** \| \| \| no elective nodal RT \| 2 \| 29 \| n.a. \| n.a. \| \| bilateral inguinal nodes \| 5 \| 71 \| 47.5 (39.6-54) \| 1.8 (1.65-2) \| \| bilateral internal iliac nodes \| 4 \| 57 \| 46.5 (39.6-50) \| 1.8 (1.65-2) \| \| bilateral external iliac nodes \| 3 \| 43 \| 46.5 (39.6-50) \| 1.8 (1.7-2) \| \| bilateral common iliac nodes \| 2 \| 29 \| 48 (39.6-50) \| 1.8 (1.7-2) \| \| mesorectal nodes \| 5 \| 71 \| 47.5 (39.6-54) \| 1.85 (1.8-2) \| \| presacral nodes \| 4 \| 57 \| 47 (39.6-52) \| 1.85 (1.8-2) \| \| bilateral obturator nodes \| 3 \| 43 \| 46.5 (39.6-50) \| 1.8 (1.65-2) \|   n: number of centers who would include the region, %: percentage of centers who would include the region, total dose: median total dose for this region (range of doses for this region), if a center specified a dose range, the mean of the dose range was used for calculation of the median values , n.a.: not applicable |
| --- | --- | --- | --- | --- | --- | --- | --- | --- | --- | --- | --- | --- | --- | --- | --- | --- | --- | --- | --- | --- | --- | --- | --- | --- | --- | --- | --- | --- | --- | --- | --- | --- | --- | --- | --- | --- | --- | --- | --- | --- | --- | --- | --- | --- | --- | --- | --- | --- | --- | --- |

**Discussion**

Definitive CRT is the standard of care for the treatment of locoregionally confined anal cancer. Although implemented for decades and evaluated in a variety of randomized phase III trials covering different aspects of the treatment, many details are still a matter of debate. Regarding radiation treatments, Austria offers a good opportunity to evaluate the pattern of care because of its “centralized” health care for roughly nine million inhabitants with only 14 centers covering radiation therapy. As a consequence, the professional community presents with manageable size and shows a high degree of commitment towards national projects. Hence, with a response rate of 93%, the present survey represents a complete and valid image of the overall practice, clearly outruling values reached in other countries. For example, a recent german survey dealing with the same subject reported only a 28% response rate [6]. We evaluated areas of agreement and disagreement between the centers and compared the general approach with the recommendations of major international guidelines, namely NCCN [2] and ESMO-ESSO-ESTRO (ESMO) [3] in its latest version.

Work-Up

There is considerable agreement on most work-up and staging issues. Multidisciplinary discussion prior to treatment is routinely done in the vast majority of institutions. All institutions routinely perform physical examination, pelvic MRI, endoscopy with biopsy and imaging of abdomen and chest, which is in line with international guidelines [2,3]. As recommended by both guidelines, chest/abdomen imaging is mainly done by CT. Interestingly, 54% of the centers indicated the use of a complete colonoscopy, although not recommended according to NCCN [2] and not required according to ESMO guidelines [3]. Same is true for endorectal ultrasound (39%), which is not routinely required according to NCCN guidelines [2]. In contrast, PET-CT is used for staging only in a minority of centers (39%), although recommended by NCCN [2] and ESMO [3] guidelines (if available) for possible advantages in staging accuracy and target volume delineation. A recent meta-analysis showed high sensitivity (93%) and specificity (76%) rates for the detection of lymph node involvement [7], which lead to upstaging in 5-38% and to downstaging in 8-27% of the patients. A systematic review further reported PET-CT to change nodal status and TNM stage in 21% and 41%, respectively [8]. Moreover, additional information provided by PET-CT may alter target volumes or dose prescription with a potential impact on outcome especially in the era of high-precision radiotherapy techniques. Mahmud et al. [7] described altered treatment plans mainly in terms of dose or field changes in 13-59% of the patients in their meta-analysis. A recent systematic review [9] reported changes just in target volume definition in 23% of the patients by the addition of PET-CT.

HIV testing should be performed in case of unknown HIV status according to NCCN [2] and ESMO [3] guidelines because the risk of anal carcinoma is markedly increased in patients bearing HIV [10] or other causes of immunosuppression [11]. However, only 15% of the Austrian centers indicated routine HIV testing, which is even lower than the 27% rate reported in the recent german survey [6]. This might be due to the fact that according to Austrian regulations, HIV testing requires a special informed consent form or because most centers would not change their treatment concept irrespective of HIV status. Retrospective data indeed indicates no clear differences in oncological outcome comparing HIV positive and negative patients and showed that changes in treatment intensity are usually not required [12,13] especially in patients adequately treated with modern antiretroviral therapy.

There is growing evidence that HPV positivity is a favorable predictive marker for locoregional control and overall survival in anal cancer patients [14-16]. NCCN [2] and ESMO [3] guideline both generally support developments in the direction of personalized medicine based on biomarkers, but to date none of the guidelines recommends routine testing of tissue samples for HPV or treatment decisions based on HPV status. The ESMO guideline in addition explicitly mentions the currently insufficient robustness of biomarkers to guide prognosis or select treatment. Accordingly, only 54% of the Austrian centers indicated to perform routine testing for HPV in biopsy samples and only one center indicated an influence of the HPV status on treatment decisions. While the large absolute differences in outcome according to HPV status in most series and the experiences from other tumor entities like oropharyngeal cancer highlight a possible benefit for tailored treatments according to HPV status, current clinical evidence seems not mature enough to convince most centers of personalized approaches (a view supported by the guideline recommendations).

Good accordance with major international guidelines was found regarding the recommendations for gynecological examinations and counseling for potential infertility. More than three quarters of the centers (77%) offered gynecological examinations either routinely or at least in case of suspected vaginal involvement to their female patients. The same proportion of institutions offered special counseling regarding possible treatment-related infertility including cryopreservation for both genders as strongly recommended by NCCN [2] and ESMO [3] guidelines.

Interestingly, only 15% of the centers perform biopsies (fine needle or excisional) in case of suspicious (clinically or on imaging) inguinal lymph nodes, although this practice is recommended by the NCCN [2] and ESMO [3] guidelines, while inguinal ultrasound as an additional staging modality is used by 23% of the institutions, although not recommended in either guideline. This practice might be explained either by the absence of clear evidence regarding improved staging accuracy with biopsies of inguinal lymph nodes compared to MRI and/or PET-CT imaging alone, or by the fact that most centers indicated to use dose escalation in LN suspicious on imaging (see below). Other reasons might be assumed treatment delay of fear and/or wound healing problems.

**Simulation/Treatment planning/treatment procedure**

Detailed recommendations specifically dealing with radiation therapy treatment planning are given to some extent within international guidelines. Supine treatment position is preferred by NCCN [2] and ESMO [3] guidelines, while prone position is advocated only based on individual decisions in both. The latter is also true for the use of bolus material. The use of intravenous and/or oral contrast-enhanced-CT for treatment planning and marking of the lower tumor edge is recommended only by the NCCN [2] guideline.

Austrian centers are mainly in line with these recommendations. All but one (92%) indicated to perform radiation therapy for anal cancer generally in supine position and bolus material is used only by a minority. While marking of the lower tumor edge is preferred by the vast majority (77%), the use of intravenous and/or oral contrast-enhanced planning CT is not (38%), although equally recommended by the NCCN [2]. All centers advise their patients to have a full bladder during treatment planning and delivery as recommended by NCCN [2]. Two thirds also advise their patients to have an empty rectum, although no specific recommendations regarding this issue are made by major guidelines. This practice is probably based on clinical experience rather than specific data, indicating that at least approximately defined organ fillings result in a more stable anatomy which limits interfraction motion.

While most high-level evidence on treatment efficacy and toxicity is based on trials using 3D-conformal radiation therapy or even 2D-techniques [4,17-20], several planning studies and retrospective comparisons indicated clear benefits in terms of sparing OARs for intensity-modulated techniques [21-23]. This advantage has been confirmed in RTOG 0529 [5], which demonstrated significantly lower grade 2+ acute hematologic, grade 3+ gastrointestinal and skin toxicities with the use of IMRT in an intended cross study comparison with data from RTOG 98-11 [24]. Therefore international guidelines [2,3] clearly recommend the use of intensity-modulated techniques, which has been adopted by all Austrian centers. Similarly, daily kv-image-guidance is advocated by the NCCN [2] guideline, which is also preferred by the majority of Austrian centers either by cone beam CT or portal imaging.

**Target volume definition and Dose prescription**

Although crucial, international guidelines reveal amazing differences in dimension and content of details concerning target volume and dose prescription. The European ESMO guideline [3] includes only very general advice regarding the target volume and literally states that specific dose recommendations cannot be made based upon currently available data. In contrast, the NCCN guideline [2] provides the readers with specific dose ranges for different disease stages as well as detailed recommendations regarding the target volume, including even two possible shrinking field techniques. These prescriptions are either based on trials using 3D-conformal RT, or specifically on RTOG 0529 [5] using IMRT with a simultaneous integrated boost technique. Therefore, guideline adherence is discussed mainly with regard to the NCCN guideline [2] in the following chapter if not otherwise stated.

Regarding total dose to the primary tumor, the NCCN guideline [2] recommends a boost beyond the doses prescribed for elective nodal areas of 5.4 to 14.4 Gy depending on T stage, resulting in total doses of 50.4 Gy to 59.4 Gy (shrinking field) or 50.4 Gy to 54 Gy (SIB-techniques). In contrast, the ESMO guideline [3] does not even give a clear recommendation for the necessity of a boost *per se*, but states that boost doses of 15-25 Gy might be prescribed to more advanced tumors, which would (although not explicitly mentioned) result in total doses of 60-70 Gy. The latter statement is of specific interest because it is in direct contrast to the results of the ACCORD 03 trial [20] and data from the RTOG 92-08 trial [25], which both provided no clear additional benefit for doses above 60 Gy [2].

Nearly all centers indicated to perform a boost to the primary tumor region by different techniques, with all but one using external-beam RT. Although all centers generally prefer IMRT techniques, the majority relies on sequential boosting (69%, shrinking field technique) rather than integrated boosting techniques (only 15%). Most centers further indicated to prescribe higher total doses in locally advanced primaries. Median total doses were 54.7 Gy for T1-2 and 59.4 Gy for T3-4 tumors, which are pretty much in line with the recommendations of the NCCN guideline [2]. This is of particular interest because actual randomized trials focus on the possible benefits of more personalized approaches evaluating dose de-escalation for early stage and dose escalation for advanced stages. For example, the PLATO platform (ISRCTN88455282) uses an umbrella design incorporating different phase II/III trials to evaluate either dose-deescalated (41,4 Gy in 23 fractions) additional chemoradiation after surgical excision in very early disease stages (ACT 3), the same de-escalated dose schedule compared to 50,4 Gy (28 fractions) as definitive treatment in intermediate disease stages, and dose-escalation up to 58,8 Gy or 61,6 Gy (28 fractions) for locally advanced stages. Moreover, the Eastern Cooperative Oncology Group evaluates deintensified chemoradiation in early disease stages in a randomized phase II trial (DECREASE, NCT04166318).

Regarding dose escalation to enlarged LN, the vast majority (85%) of the Austrian centers indicated the use of a boost. Most centers prescribe the boost only to the enlarged LN, while one center increases the dose in the affected lymph node region. The median total dose to an enlarged LN was 54 Gy (range 50-60 Gy). While the preferred technique of boosting only the involved node is in line with the NCCN recommendations [2], the median reported boost dose is equivalent to the dose recommended only for nodes larger than 3 cm [2]. Interestingly, only one of the centers indicated different doses depending on LN size, although recommended by the NCCN [2].

Because of a possible impact of the presence of nodal disease on target volume definition, we asked detailed questions regarding the covered elective nodal areas for both situations (cN0 vs cN1). Generally, high concordance rates between the centers regarding the covered nodal regions exist for both scenarios. Most centers (85%) will regularly include the bilateral inguinal, iliac external, iliac internal, mesorectal, presacral and obturator nodes into the elective nodal volume with an even slightly increased concordance rate for nodal-positive patients (92%). This pattern does exactly match the recommendations by the NCCN guideline [2]. The only matter of debate seems to be the common iliac node area. While in cN0 patients, after all 46% of the centers indicated the inclusion of this area, this rate even increased to 62% in cN1 patients. This finding seems somewhat surprising as neither the NCCN [2] nor the ESMO [3] guideline recommends the inclusion of the common iliac nodes in neither situation. Moreover, none of the recent phase III trials [19,20,24] nor RTOG 0529 [5] did include this area into the elective nodal volume. However, increasing evidence from retrospective pattern of failure analyses suggest that especially patients with pelvic nodal disease and/or locally advanced primary tumors may have an increased and substantial risk of positive common iliac or even lower paraaortic nodes [26-28]. Thus, their inclusion into elective nodal target volumes might be justified at least in the presence of such risk factors. Adherence to international target volume guidelines or evaluation of interobserver variability in case examples would be of further interest, but was beyond the scope of the survey.

Prescription doses to elective nodal volumes distinctly differ between the major trials [5,19,20,24], although all prescribed doses in the range of 30.6 to 45 Gy, thus making specific recommendations difficult. Consequently, the NCCN [2] guideline generally recommends this dose range (if a shrinking field technique is used), but includes a distinction into so-called low-risk and high-risk elective nodal volumes, which are mainly separated by the inferior border of the sacroiliac joint. While the former should be treated with doses at the lower end of the recommended range, the latter should be treated with doses at the upper end. For patients treated with integrated boost concepts, the NCCN [2] guideline recommends total doses of 42-45 Gy in 1.5 Gy per fraction (equivalent to roughly 40-42 Gy in conventional fractionation assuming an alpha/beta of 10) to all elective nodal regions based on RTOG 0529 [5]. In contrast, the ESMO [3] guideline just recommends to include “any sites of likely nodal involvement” with no specific dose recommendation.

All centers indicated doses to elective nodal regions within the recommended dose ranges of both guidelines or above. The reported median doses are somewhat lower for cN0 patients and higher for cN+ cases, indicating the assumption of a generally higher risk for subclinical disease in cN+ patients and the idea to control this subclinical disease with a slightly increased dose. The median reported doses are above the recommended range especially for cN+ patients (50-50.4 Gy) and to a lesser extent for cN0 patients, although the value of this dose escalation has not been evaluated or proven by randomized trials. Moreover, the majority of centers does not follow the distinction into low risk and high risk regions as recommended by the NCCN [2] guidelines, but prescribes a uniform dose to all included elective nodal regions instead.

Regarding dose constraints for OAR, only the NCCN [2] (of the mentioned) guidelines provide specific recommendations if intensity-modulated RT with integrated boost concepts are used. The answers given by the Austrian centers showed a large diversity of generally considered OARs and large variations in accepted doses. While bladder, femoral heads and bowel structures are at least outlined in the majority of centers, this was not the case for external genitalia or pelvic bone. The use of specific constraints was reported only for bladder and femoral heads in the majority of centers. It seemed that many of the reported constraints were not specific for anal cancer but derived from treatment for less rare tumors like prostate or rectal cancer (some of the reported constraints of the centers were higher than the total doses indicated for the primary tumor boost). Contouring OARs and using specific dose constraints for anal cancer might be a field of further improvement.

**Systemic chemotherapy**

Very strong agreement among all Austrian centers and accordance to international guidelines exists regarding the chemotherapy regimens for simultaneous CRT. All institutions indicated the use of a doublet including Mitomycin C (MMC) and 5-FU or capecitabine. None of the centers favors a Cisplatin-based regime. This may reflect the results of the two largest trials comparing MMC/5-FU with Cisplatin/5-FU for simultaneous CRT [19,24], which both showed benefits for the MMC/5-FU combination either regarding oncological outcome [24] or toxicity [19]. Capecitabine is preferred over infusional 5-FU by the slight majority of centers, which may either reflect its easier application especially during out-patient treatment or because retrospective data suggests lower hematological toxicity with capecitabine compared to infusional 5-FU in patients reciving IMRT [29]. While the centers favoring capecitabine even agreed on the same dose schedule, slight differences between the centers using infusional 5-FU have been reported, although nearly all follow dose prescriptions recommended by major guidelines [2,3] and supported by major trials [5,19,22].

**Supportive care**

Most centers perform CRT mainly on an out-patients basis, although 31% indicated to regularly treat their patients on their wards. This may reflect either the assumption of a need for intensified care of treatment side effects like skin reactions, bowel dysfunction and hematological complications or the lack of out-patient structures to assure adequate care for those common toxicities. Another reason may be the somewhat “centralized” structure of radiation oncology care in Austria, which sometimes results in high travel distances (or at least long travel durations) especially in the mountainous regions of the country.

Clear recommendations regarding supportive care for specific acute or late reactions are rarely supported by high-level evidence and consequently rarely provided by any of the major anal cancer guidelines. However, clear differences between the guidelines exist regarding the extent of information on this issue in general. While the European ESMO [3] guidelines includes only a very short paragraph on this subject, the NCCN [2] guideline directly refers to another guideline (NCCN principles of survivorship [30]), which provides detailed general recommendations although based on symptoms rather than tumor entity or treatment.

To cover this complex subject, we had asked two questions referring to issues that are generally recommended by both guidelines independent of the treatment (namely nutritional counseling and psycho-oncological support [3,30] and one question referring to late toxicity care (namely prevention of vaginal stenosis in females). Interestingly, only a minority of centers offers nutritional advice (38%) or psycho-oncological support (23%) on a regular basis, although both issues affect the majority of surviving patients after pelvic radiation therapy according to QoL studies [31,32]. In contrast, 54% advised female patients to use vaginal dilatators to prevent stenosis, which represents a high level of awareness and care compared to other reports dealing with sexual dysfunction after pelvic radiation therapy [32].

**Follow-up**

Recommendations regarding follow-up investigations after chemoradiation for anal cancer distinguish between response evaluation and follow-up in case of complete remission. One of the main issues in response evaluation is the time point when to consider histological confirmation of clinically persistent disease for potential salvage surgery. Austrian centers are divided roughly equally between a three months and a six month interval, although both international guidelines [2,3] clearly favor the latter time point based on the data from ACT II [33]. In this trial, 72% of the patients with persistent disease at 11 weeks achieved a complete response by 26 weeks without further treatment [33], indicating that many salvage procedures can be avoided by increasing the time to final response evaluation.

Regarding follow-up after complete remission, major guidelines favor DRE as the mainstay of evaluation [2,3], supported by inguinal node palpation [2,3] and anoscopy [2] every 3-6 months for 5 years [2]. The NCCN [2] guideline further recommends cross-sectional imaging of chest, abdomen and pelvis annually for three years in patients with advanced disease (T3/4 or N+), while the ESMO [3] guidelines mention the necessity of addition cross sectional imaging of the pelvis but without giving recommendation on intervals or duration of follow-up. Accordingly, all Austrian institutions offer regular follow-up to their patients, although distinct differences in care provider, time intervals, extent of examinations and duration of follow-up exist. Most centers use increasing time intervals during follow-up, usually starting with 3 months intervals for the first 1-2 years, which are increased to 6-12 months over time. In contrast to the above mentioned recommendations, those visits usually include DRE, endoscopy and cross-sectional imaging (mainly pelvic MRI and chest/abdominal CT) at equal intervals. In accordance to the NCCN recommendations [2], the minimum follow-up period is 5 years in the participating centers, while 15% even increase this period to 8-10 years.

**Case: Early anal cancer: How to treat?**

The optimal treatment for very early stage anal cancer (cT1N0) is still controversial. Main treatment options include surgery and (chemo)-radiation. No randomized trials comparing these options exist.

The NCCN [2] guideline recommends CRT as the primary treatment option for early stage anal cancer in general but mentions two exceptions, in which primary surgical management may be appropriate:

1. Superficially invasive anal cancer which has been completely excised with ≤3mm basement membrane invasion and a maximal horizontal spread of ≤ 7mm

2. T1N0, well-differentiated perianal cancer or selected T2N0 perianal cancer that does not involve the sphincter

The ESMO [3] guideline mentions that T1N0 well or moderately differentiated anal margin cancers may be properly treated by primary surgery if adequate clear margins can be obtained without compromising sphincter function. However, surgery for even small tumors of the anal canal is contraindicated according to this guideline.

According to our survey (including detailed questions on a T1N0 well differentiated tumor located in the transition zone), Austrian radiation oncologist are divided regarding the general management of early stage anal cancer. Only a small majority (54%) advocated for (chemo)radiation, while many still preferred local excision. Similar results have been reported by a recent Australian survey including surgeons and radiation oncologists [34]. This may reflect a general trend to more local excisions as primary treatment for early stage anal cancer as shown by a recent National cancer database analysis [35], which further reported no difference in overall survival between local excision and CRT. However, no data on other endpoints like local control, regional control, distant metastases or side effects were reported due to the nature of this population-based analysis and two third of the patients have still received primary chemoradiation [35]. Another reason might be that many very early lesions are excised primarily for diagnostic purposes. Because no clear evidence for a need of additional treatment exists if clear margins have been achieved (even unintentionally), clinicians may have adopted this practice as a general option. However, favouring a surgical approach might be simply based on confusion over terminology between perianal cancers and cancer of the true anal canal as speculated by Jones et al. [34]

Regarding (chemo)radiation parameters, even more disagreement among Austrian radiation oncologists exist. Of seven institutions which indicated to prefer a non-surgically approach, only three would use CRT while four prefer radiation alone. Early stage patients (especially T1N0) have been excluded from almost every randomized trial evaluating CRT approaches for anal cancer including the trial which proved the benefit of adding chemotherapy to RT [4]. Thus, no clear evidence for a benefit of adding chemotherapy exists, while its increased acute toxicity compared to radiation alone is clearly proven [36]. Moreover, a recent SEER database analysis including 190 patients reported no OS difference between CRT vs radiation alone [37]. This may prompt many radiation oncologists to use radiation alone instead of the combined approach in order not to harm the patients.

Distinct differences also exist regarding the target volumes and prescribed doses. Of the seven institutions favoring CRT, five would include elective nodal regions while two would treat only the primary. In the absence of large pattern of nodal spread or pattern of failure analyses for early stage anal cancer, considerable disagreement exists regarding the nodal regions to be treated. The highest rate of agreement was found for the bilateral inguinal and the mesorectal nodes, followed by bilateral internal iliac and presacral nodes.

Interestingly, the prescribed doses to the primary tumor seemed somewhat high for early stage anal cancer (median dose 57.2 Gy), especially if compared to the doses reported for treatment of more advanced lesions, however this maybe based on the fact that the majority advocated the use of radiation alone instead of combined CRT. In contrast, the median prescribed doses to elective nodal areas are very similar to the doses reported for more advanced but node negative tumors. Equally to the more advanced scenarios, all centers favouring CRT for early stage anal cancer would use Mitomycin C and 5-FU or capecitabine as chemotherapy.

**Summary**

In summary, we found a high rate of agreement between the centers and concordance with the recommendations of major international guidelines at least covering the main issues of work-up, treatment and follow-up for CRT for anal cancer. Only PET-CT, routine HIV testing and biopsy of suspicious LN seem to be less frequently used during work-up than recommended. All centers use modern radiation techniques (IMRT) including image-guidance. While high agreement and concordance to guidelines exist in general on elective nodal target volumes, a large controversy exists regarding the inclusion of the common iliac nodes. Prescription doses to primary tumor, suspected LN and elective nodal areas vary to some extent, but are generally in line with the recommendations, although sometimes at or above the recommended upper dose range. In contrast, no agreement on delineation of OARs or dose constraints exists, which raises a possible need for standardization. Supportive care during/after CRT is highly variable and underrepresented in major guidelines, although this is assumed to be an integral part of the treatment. Follow-up is even done more intensively than recommended. Considerable disagreement regarding the treatment of early anal cancer exists, including treatment approach (surgery vs radiation alone vs CRT) as well as target volumes and prescription doses, indicating a need for further research.

**References**

**1. Nigro ND,** Vaitkevicius VK, Considine B. Combined therapy for cancer of the anal canal: a preliminary report. Dis Colon Rectum 1974;17:354-356

**2. NCCN Clinical** Practice Guidelines in Oncology Anal Carcinoma, Version 2.2020. [www.nccn.org](http://www.nccn.org). Assessed on Dec 20, 2020

**3. Glynne-Jones R,** Nilsson PJ, Aschele C, et al. Anal cancer: ESMO-ESSO-ESTRO clinical practice guidelines for diagnosis, treatment and follow up. Radiother Oncol 2014;111:330-339

**4. Northover J,** Glynne-Jones R, Sebag-Montefiore D, et al. Chemoradiation for the treatment of epidermoid anal cancer: 13-year follow-up of the first randomized UKCCR anal cancer trial (ACT I). Br J Cancer 2010;102:1123-1128

**5. Kachnic L,** Winter K, Myerson RJ, et al. RTOG 0529: A Phase II Evaluation of Dose-Painted Intensity Modulated Radiation Therapy in Combination with 5-Fluorouracil and Mitomycin-C for the Reduction of Acute Morbidity in Carcinoma of the Anal Canal. Int J Radiat Oncol Biol Phys 2013;86:27-33

**6. Martin D,** von der Grün J, Rödel C, Fokas E. Management of anal cancer patients – a pattern of care analysis in german-speaking countries. Radiat Oncol 2020;15:22

**7. Mahmud A,** Poon R, Jonker D. PET imaging in anal cancer: a systematic review and meta-analysis. Br J Radiol 2017;90:20170370

**8. Jones M,** Hruby G, Solomon M, et al. The role of FDG-PET in the initial staging and response assessment of anal cancer: a systematic review and meta-analysis. Ann Surg Oncol 2015;22:3574-3581

**9. Albertsson P,** Alverbratt C, Liljegren A, et al. Positron emission tomography and computed tomography (PET/CT) imaging for radiation therapy planning in anal cancer: a systematic review and meta-analysis. Crit Rev Oncol Hematol 2018;126:6-12

**10. Patel P,** Hanson DL, Sullivan PS, et al. Incidence of types of cancer among HIV-infected persons compared with the general population in the United States, 1992-2003. Ann Intern Med 2008;148:728-736

**11. Grulich AE,** van Leeuwen MT, Falster MO, Vajdic CM. Incidence of cancers in people with HIV/AIDS compared with immunosuppressed transplant recipients: a meta-analysis. Lancet 2007;370:59-67

**12. Fraunholz I,** Weiss C, Eberlein K, et al. Concurrent chemoradiotherapy with 5-fluorouracil and mitomycin c for invasive anal carcinoma in human immunodeficiency virus-positive patients receiving highly active antiretroviral therapy. Int J Radiat Oncol Biol Phys 2010;76:1425-1432

**13. Fraunholz IB,** Haberl A, Klauke S, et al. Long-term effects of chemoradiotherapy for anal cancer in patients with HIV infection: oncological outcomes, immunological status and the clinical course of the HIV disease. Dis Colon Rectum 2014;57:423-431

**14. Urbute A**, Rasmussen CL, Belmonte F, et al. Prognostic significance of HPV DNA and p16^INK4a^ in anal cancer: a systematic review and meta-analysis. Cancer Epidemiol Biomarkers Prev 2020;29:703-10

**15. Balermpas P**, Martin D, Wieland U, et al. Human papilloma virus load and PD-1/PD-L1, CD8(+) and FOXP3 in anal cancer patients treated with chemoradiotherapy: rationale for immunotherapy. Oncoimmunology 2017;6:e1288331

**16. Rödel F**, Wieland U, Fraunholz I, et al. Human papillomavirus DNA and p16^INK4a^ expression predict for local control in patients with anal squamous cell carcinoma treated with chemoradiotherapy. Int J Cancer 2015;136:278-288

**17. Flam M,** John M, Pajak TF, et al. Role of mitomycin in combination with fluorouracil and radiotherapy, and of salvage chemoradiation in the definitive nonsurgical treatment of epidermoid carcinoma of the anal canal: results of a phase III randomized intergroup study. J Clin Oncol 1996;14:2527-2539

**18. Ajani JA,** Winter KA, Gunderson LL, et al. Fluorouracil, mitomycin, and radiotherapy vs fluorouracil, cisplatin, and radiotherapy for carcinoma of the anal canal: a randomized controlled trial. JAMA 2008;299:1914-1921

**19. James RD,** Glynne-Jones R, Meadows H, et al. Mitomycin or cisplatin chemoradiation with or without maintenance chemotherapy for treatment of squamous-cell carcinoma of the anus (ACT II): a randomized, phase 3, open-label, 2x2 factorial trial. Lancet Oncol 2013;14:516-524

**20. Pfeiffert D,** Tournier-Rangeard L, Gerard JP, et al. Induction chemotherapy and dose intensification of the radiation boost in locally advanced anal canal carcinoma: final analysis of the randomized UNICANCER ACCORD 03 trial. J Clin Oncol 2012;30:1941-1948

**21. Chen YJ,** Liu A, Tsai P, et al. Organ sparing by conformal avoidance intensity-modulated radiation therapy for anal cancer: dosimetric evaluation of coverage of pelvis and inguinal/femoral nodes. Int J Radiat Oncol Biol Phys 2005;63:274-281

**22. Chuong MD,** Freilich JM, Hoffe SE, et al. Intensity-modulated radiation therapy vs. 3D conformal radiation therapy for squamous cell carcinoma of the anal canal. Gastrointest Cancer Res 2013;6:39-45

**23. Sauter M,** Lombriser N, Bütikofer S, et al. Improved treatment outcome and lower skin toxicity with intensity-modulated radiotherapy vs. 3D conventional radiotherapy in anal cancer. Strahlenther Onkol (2020) 196:356–367

**24. Gunderson LL,** Winter KA, Ajani JA, et al. Long-term update of US GI Intergroup RTOG 98-11 phase III trial for anal carcinoma: survival, relapse, and colostomy failure with concurrent chemoradiation involving fluorouracil/mitomycin versus Fluororuracil/cisplatin. J Clin Oncol 2012;30: 4344-4351

**25. John M,** Pajak T, Flam M, et al. Dose escalation in chemoradiation for anal cancer: preliminary results of RTOG 92-08. Cancer J Sci Am 1996;205-211

**26. Wright JL,** Patil SM, Temple LK, et al. Squamous cell carcinoma of the anal canal: patterns and predictors of failure and implications for intensity-modulated radiation treatment planning. Int J Radiat Oncol Biol Phys 2010;78:1064-1072

**27. Nilsson MP,** Nilsson ED, Johnsson A, et al. Patterns of recurrence in anal cancer: a detailed analysis. Radiat Oncol 2020;15:125

**28. Dapper H,** Schiller K, Münch S, et al. Have we achieved adequate recommendations for target volume definitions in anal cancer ? A PET imaging based patterns of failure analysis in the context of established contouring guidelines. BMC cancer 2019;19:742

**29. Goodman KA,** Julie D, Cercek A, et al. Capecitabine with Mitomycin reduces acute hematologic toxicity and treatment delays in patients undergoing definitive chemoradiation using intensity modulated radiation therapy for anal cancer. Int J Radiat Oncol Biol Phys 2017;98:1087-1095

**30. NCCN Clinical** Practice Guidelines in Oncology Survivorship, Version 2.2020,ww.nncn.org, assessed on Dec 20, 2020

**31. Sunesen KG,** Norgaardt M, Lundby L, et al. Long-term anorectal, urinary and sexual dysfunction causing distress after radiotherapy for anal cancer: a danish multicentre cross-sectional questionnaire study. Colorect Dis 2015;17:O230-239

**32. White ID,** Allan H, Faithfull S. Assessment of treatment-induced female sexual morbidity in oncology: is this a part of routine medical follow-up after radical pelvic radiotherapy ? Br J Cancer 2011;105:903-910

**33. Glynne-Jones R,** Sebag-Montenfiori D, Meadows HM, et al. Best time to assess complete clinical response after chemoradiotherapy in squamous cell carcinoma of the anus (ACT II): a post-hoc analysis of randomized controlled phase III trial. Lancet Oncol 2017;18:347-356

**34. Jones MP,** Caroll S, Martin J, et al. Management of early stage anal cancer: need for guidelines and standardization. Int J Colorectal Dis 2017;32:1719-1724

**35. Chai CY,** Cao HT, Awad S, Massarweh N. Management of stage I squamous cell carcinoma of the anal canal. JAMA Surg 2018;153:209-215

**36. UKCCCR Anal Cancer trial working party. Epidermoid anal cancer: results from the UKCCCR randomized trial of radiotherapy alone versus radiotherapy, 5-fluorouracil, and mitomycin. Lancet 1996;348:1049-1054**

**37. Desmukh AA,** Zhao H, Das P, et al. Clinical and economic evaluation of treatment strategies for T1N0 anal cancer. Am J Clin Oncol 2018;41:626-631
