# Supplementary material for: Practice of radiation therapy for anal cancer in Austria—a survey on behalf of the Austrian radiation oncology society gastrointestinal tumor group (ÖGRO-GIT)
Source: Strahlenther Onkol. 2021 Sep 30;197(11):953–61. doi: 10.1007/s00066-021-01842-w (PMC8547205; doi:10.1007/s00066-021-01842-w)
Supplement: Supplementary file 2 — Questions [file 66_2021_1842_MOESM2_ESM.docx]

**Allgemeine Fragen**

1. Wie viele Linearbeschleuniger werden bei Ihnen betrieben?

- 1-3
- 4-6
- >6

1. Gehört Ihre Abteilung einem Universitätsklinikum an?

- Ja
- Nein

1. Wie viele Patienten mit Analkarzinom in der Primärsituation werden in Ihrer Institution pro Jahr in etwa strahlentherapeutisch behandelt?

____________

1. Gibt es an Ihrem Zentrum offene klinisch-prospektive Studien für Patienten mit Analkarzinom mit radioonkologischer Beteiligung?

- Ja
- Nein

Wenn ja, welche?

__________________________________________________________

__________________________________________________________

__________________________________________________________

1. Liegen in Ihrer Institution standardisierte schriftliche Verfahrensanweisungen für die radioonkologische Behandlung von Analkarzinomen vor (SOPs)?

- Ja
- Nein

**Definitive RCT**

**Indikationsstellung:**

1. Erfolgt eine regelhafte prätherapeutische Vorstellung im interdisziplinären Tumorboard?

- Ja, praktisch immer
- Ja, meistens (> 80% der Fälle)
- überwiegend (50-80% der Fälle)
- eher selten (30-50% der Fälle)
- kaum (weniger als 30% der Fälle)

1. Welche Voruntersuchungen werden zur Therapievorbereitung regelhaft (> 80% der Fälle) durchgeführt? (bitte Mehrfachauswahl)

- DRE
- Leistensonographie
- Oberbauchsonographie
- CT Abdomen/Becken
- Rö-Thorax
- CT Thorax
- Coloskopie
- Proktoskopie/Rectoskopie/Sigmoidoskopie
- Endosonographie
- MRT Becken
- FDG – PET CT
- Sonstige:_______________________________________________

1. Enthält die histopathologische Untersuchung standardmäßig den HPV 16/18 Status?

- Ja
- Nein

1. Wenn ja, beeinflusst eine HPV Positivität das Therapieregime?

- Ja
- Nein

1. Werden suspekte Leistenlymphknoten regelhaft biopsiert?

- Ja
- Nein

1. Erfolgt vor der Therapieeinleitung regelhaft ein HIV Test des Patienten/innen?

- Ja
- Nein

1. Wenn ja, beeinflusst das Ergebnis regelhaft das Therapieregime?

- Ja
- Nein
- Wenn ja, inwiefern: ________________________

1. Erfolgt bei weiblichen Patientinnen regelhaft eine prätherapeutische gynäkologische Untersuchung?

- Ja
- Nein
- Nur bei Vorliegen von: ___________________

14. Erfolgt bei Patienten/Patientinnen mit Kinderwunsch regelhaft eine explizite Aufklärung über die Möglichkeit einer Kryokonservierung?

- Ja
- Nein

**Durchführung RTx/RCT**

1. Wie wird der/die Patient/in üblicherweise für die Radiotherapie gelagert?

- Rückenlage
- Bauchlage

1. Welche Lagerungs-/Planungsmaßnahmen führen Sie regelhaft durch?

(bitte Mehrfachauswahl)

- Markierung/Distanzierung der Vagina z.B. mittels Tampon
- Markierung des Anus (anokutaner Übergang) bzw. des Tumorunterrrandes (bei Prolaps)
- Blasenfüllung: volle Blase leere Blase definiertes Blasenvolumen keine Vorgabe
- Rektumfüllung: leeres Rektum keine Vorgabe

1. Erfolgt das Planungs-CT mit oralem Kontrastmittel?

- Nein
- Ja, wenn keine Kontraindikationen

1. Erfolgt das Planungs-CT mit i.v. Kontrastmittel?

- Nein
- Ja, wenn keine Kontraindikationen

1. Verwenden Sie Bolusmaterial beim Planungs-CT?

- ja, anal immer
- ja, anal bei prolabierendem Tumor
- ja, inguinal immer
- ja, inguinal bei suspekten/gesichert befallenen oberflächlichen LK
- nein
- Wenn ja, welches Material: ______________________

Die Fragen 20-22 betreffen die Zielvolumendefinition sowie die verschriebenen Gesamt- bzw. Einzeldosen in verschiedenen klinischen Situationen.

In Frage 20 soll die Ausdehnung und Dosisverschreibung bzw. Fraktionierung der elektiven Lymphabflusswegbestrahlung bei Patienten mit klinischer/bildmorphologischer N0 Situation angegeben werden. In Frage 21 sollen die gleichen Angaben für Patienten mit bildmorphologisch oder histologisch gesichertem LK-Befall erfolgen, wiederum jedoch bezogen auf die elektiven (nicht-befallenen) Regionen. In Frage 22 wird das Vorgehen bezogen auf die befallene(n) LK-(Region) abgefragt. Hier soll zum einem angegeben werden ob ein befallener LK selbst oder eine befallene LK-Region mit einer höheren Dosis belegt wird als in Frage 21 (cN+-Situation) angegeben. Weiterhin soll angegeben werden ob das bei jedem bildmorphologisch/histologisch befallenen LK erfolgt oder erst ab einer bestimmten Größe des LK.

Sollte Ihre Vorgehensweise sich gar nicht oder nur teilweise abbilden lassen, bitten wir um eine möglichst präzise Freitextbeschreibung (siehe Textfeld nach Frage 22)

1. Zielvolumendefinition elektive Lymphabflusswege bei cN0 Situation (Primärtumor > T1): Welche Regionen schließen Sie ein? Bei Einschluss bitte die jeweilige Gesamtdosis und Einzeldosis angeben (bitte Mehrfachauswahl).

- LAW inguinal bds. Gesamtdosis: _____ Einzeldosis: _____
- LAW iliacal intern bds. Gesamtdosis: _____ Einzeldosis: _____
- LAW iliacal extern bds. Gesamtdosis: _____ Einzeldosis: _____
- LAW iliacal commun bds. Gesamtdosis: _____ Einzeldosis: _____
- LAW präsacral Gesamtdosis: _____ Einzeldosis: _____
- LAW mesorectal Gesamtdosis: _____ Einzeldosis: _____
- LAW obturatorisch bds. Gesamtdosis: _____ Einzeldosis: _____
- sonstige:_____________ Gesamtdosis: _____ Einzeldosis: _____
- Kein elektiver Lymphabfluss

1. Zielvolumendefinition elektiver Lymphabflusswege bei cN+ Situation: Welche Regionen schließen Sie ein? Bei Einschluss bitte die jeweilige Gesamtdosis und Einzeldosis angeben (bitte Mehrfachauswahl).

- LAW inguinal bds. Gesamtdosis: _____ Einzeldosis: _____
- LAW iliacal intern bds. Gesamtdosis: _____ Einzeldosis: _____
- LAW iliacal extern bds. Gesamtdosis: _____ Einzeldosis: _____
- LAW iliacal commun bds. Gesamtdosis: _____ Einzeldosis: _____
- LAW präsacral Gesamtdosis: _____ Einzeldosis: _____
- LAW mesorectal Gesamtdosis: _____ Einzeldosis: _____
- LAW obturatorisch bds. Gesamtdosis: _____ Einzeldosis: _____
- sonstige:_______________ Gesamtdosis: _____ Einzeldosis: _____
- Kein elektiver Lymphabfluss

1. Bei befallenen Lymphknoten: Erhöhen Sie die Dosis im befallenen LK selbst (LK-Boost) oder in der gesamten zugehörigen LK-Region des befallenen LK, wenn ja welche Gesamtdosis und Einzeldosis streben Sie an:

- einzelnen befallenen LK (LK-Boost) Gesamtdosis: _____ Einzeldosis: ____
- der gesamten betroffenen LK-Region Gesamtdosis: _____ Einzeldosis: ____
- gar nicht

Gehen Sie bei allen suspekten/gesicherten LK so vor oder erst ab einer bestimmten Größe, wenn ja ab welcher Größe?

- bei allen suspekten/gesicherten
- nur bei LK > __________ cm

Freitext für nicht-abbildbares Konzept zu den Fragen 20-22:

1. Wird regelhaft ein Boostvolumen auf den Tumor definiert?

- Ja
- Nein

1. Wenn ja, welche Dosis streben Sie bei T1/T2 Tumoren an?

Gesamtdosis: _____ Einzeldosis: ____

1. Wenn ja: welche Dosis streben Sie bei T3/T4 Tumoren an?

Gesamtdosis: _____ Einzeldosis: ____

1. Wie erfolgt die Boostapplikation auf den Primärtumor?

- perkutan simultan-integriert (dose-painting)
- perkutan sequenziell
- Elektronenboost
- Brachytherapieboost

1. Für welche Organe geben Sie Constraints vor? Wenn ja welche? Falls keine allge -meinen Constraints vorgegeben werden aber die Belastung regelhaft im Planungs - prozess beachtet wird bitte ALARA eintragen. (bitte Mehrfachauswahl) ALARA bei der Benennung der einzelnen Organe rausnehmen

- Blase:___________________
- Bowelbag (*):_____________
- Dünndarm: _______________
- äußeres Genitale:__________
- Colon: ___________________
- Beckenknochen: ___________
- Hüftköpfe: ________________
- Cauda equina: _____________
- Sonstige:__________________

*(Definition nach RTOG: Peritoneal space occupied or potentially occupied by bowel, large or small, siehe Int J Radiat Oncol Biol Phys 2012;83:e353-362)

1. Die Radiotherapie erfolgt regelhaft mittels

- 3–D konformaler RT
- step and shoot IMRT
- volumetrische IMRT
- helikale IMRT
- Sonstiges:______________

1. Wie erfolgt die IGRT üblicherweise (keine außergewöhnlichen Lagerungsunge - nauigkeiten oder Patientenverhältnisse vorausgesetzt)? (Mehrfachauswahl bei Kombinationen möglich)

- Täglich mittels Kontrollaufnahmen/Portal Imaging
- 2-3x/Woche mittels Kontrollaufnahmen/Portal Imaging
- 1x /Woche mittels Kontrollaufnahmen/Portal Imaging
- Täglich mit CBCT
- 2-3x /Woche CBCT
- 1x /Woche CBCT
- 3-5 Kontrollen (CBCT) am Anfang: danach je nach Mittelwertverschiebung
- 3-5 Kontrollen (Portal Imaging) am Anfang: danach je nach Mittelwert -verschiebung
- anderes Schema/andere Technik: ________________________________
- zusätzlich Ultraschallgesteuerte Messung der Blasenfüllung

**Simultane Chemotherapie:**

1. Welche Chemotherapie applizieren Sie standardmäßig?

- 5 FU/MMC
- Capecitabine/MMC
- 5 FU/Cisplatin
- Capecitabine/Cisplatin
- MMC/Cisplatin
- andere Kombination: ________________
- Monotherapie mit __________________

1. In welcher Dosierung/zeitlichen Abfolge geben Sie die Chemotherapie?

(z.B. MMC 10 mg d1, 5-FU 1000 mg d1-4 Wdh d28)

______________________________________________________________

______________________________________________________________

1. Wird die Chemotherapie von ihnen/in der Strahlentherapie appliziert?

- Ja
- Nein
- Wenn Nein, wer appliziert die Chemotherapie:________________________

**Supportivtherapie**

1. Welche supportiven Massnahmen (nicht Haut/Schleimhautpflege) erfolgen regelhaft? (Mehrfachauswahl möglich)

- Ernährungsberatung
- Psychoonkologie
- Vaginalstenoseprophylaxe
- Sonstiges:______________________

1. Erfolgt die Radiochemotherapie unter stationären Bedingungen?

- Ja, überwiegend und regelhaft
- Ja, teilweise (nur zur Chemotherapie und/oder bei akuter Beschwerdesympto -matik)
- Nein

**Nachsorgestrategie:**

1. Wo erfolgt die Nachsorge? (Mehrfachauswahl möglich)

- Onkologie im Hause
- Radioonkologie im Hause
- Gastroenterologie im Hause
- niedergelassener/zuweisender Onkologe
- niedergelassener/zuweisender Gastroenterologe
- Hausarzt
- Sonstige:________________________
- keine
- In Welchen Zeitabständen erfolgt die Nachsorge (z.B. alle 3 Monate)?
- Im 1. Jahr: _______________
- Im 2. Jahr: _______________
- Ab dem 3. Jahr: _______________
- Anderes Schema:______________
- Wie viele Jahre wird sie regelhaft fortgeführt? _____________

1. Welche Untersuchungen beinhaltet die Nachsorge regelhaft (Mehrfachauswahl)

- DRE
- Leistensonographie
- Rö-Thorax
- CT Thorax
- Oberbauchsonographie
- CT Abdomen/Becken
- Coloskopie
- Proktoskopie/Rectoskopie/Sigmoidoskopie
- Endosonographie
- MRT Becken
- FDG – PET CT
- Lebensqualität/Qol Bögen
- Sonstiges:_______________________________________________

1. Bei Verdacht auf (bildmorphologisch/endoskopisch nicht progredienten oder regre- dienten) Tumorpersistenz nach Therapie: wann empfehlen Sie frühestens eine histo -logische Probeentnahme?

- Nach 4-6 Wochen
- Nach 3 Monaten
- Nach 6 Monaten
- sonstige:__________________

**Spezialfall frühes Analkarzinom, Indikation und Therapie:**

1. 50 - Jährige Patientin, keine relevanten Komorbiditäten, Tumorlokalisation Übergang Analschleimhaut zu Analhaut, cT1 cN0 cM0, G1; Welche Therapie schlagen Sie vor?

- Locale Exzision
- Abdomino-perineale Resektion
- Radiotherapie
- Radiochemotherapie
- sonstige:____________________

Wenn Sie sich für eine Radiotherapie/Radiochemotherapie entscheiden:

Welche Dosis für den elektiven Lymphabflusses würden Sie applizieren?

- Gesamtdosis: __________, Einzeldosis _______________
- Die Radiotherapie erfolgt ohne elektiven Lymphabfluss

Welche Dosis für den Primärtumor würden Sie applizieren?

- Gesamtdosis: __________, Einzeldosis _______________

Welche Systemtherapie würden Sie applizieren?

- MMC/5-FU
- MMC/Capecitabine
- Cisplatin/5-FU
- Cisplatin/Capecitabine
- andere Kombination: ______________________
- Monotherapie mit ______________________
- Dosisreduktion der üblichen Dosierung der genannten Systemtherapie auf _______________%
- keine

Wenn Sie sich für eine Radio(chemo)therapie entscheiden: welche Regionen schliessen Sie ein? (bitte Mehrfachauswahl)

- Tumor/erw. Tumorregion Gesamtdosis____________ Einzeldosis _______
- LAW inguinal bds. Gesamtdosis____________ Einzeldosis _______
- LAW iliacal intern bds. Gesamtdosis____________ Einzeldosis _______
- LAW iliacal extern bds. Gesamtdosis____________ Einzeldosis _______
- LAW iliacal commun bds. Gesamtdosis____________ Einzeldosis _______
- LAW präsacral Gesamtdosis____________ Einzeldosis _______
- LAW mesorectal Gesamtdosis____________ Einzeldosis _______
- LAW obturatorisch bds. Gesamtdosis____________ Einzeldosis _______
- sonstige:_____________ Gesamtdosis____________ Einzeldosis _______
